# Stratified sustainable vector control strategies and measures for malaria control and elimination in China: a 70 year journey

**DOI:** 10.1136/bmj-2024-080656

**Published:** 2025-04-22

**Authors:** Qiyong Liu, Yiguan Wang, Xiaobo Liu, Simon I Hay, Shengjie Lai

**Affiliations:** 1 National Key Laboratory of Intelligent Tracking and Forecasting for Infectious Diseases, National Institute for Communicable Disease Control and Prevention, Chinese Center for Disease Control and Prevention; WHO Collaborating Centre for Vector Surveillance and Management, Beijing, China; 2 Department of Vector Control, Department of Epidemiology, School of Public Health, Cheeloo College of Medicine, Shandong University, Jinan, Shandong province, China; 3 CAS Key Laboratory of Insect Developmental and Evolutionary Biology, CAS Center for Excellence in Molecular Plant Sciences, Shanghai, China; 4 Department of Health Metrics Sciences, School of Medicine, University of Washington, Seattle, WA, USA; 5 WorldPop, School of Geography and Environmental Science, University of Southampton, Southampton, UK; 6 Institute for Life Sciences, University of Southampton, Southampton, UK

## Abstract

**Qiyong Liu and colleagues** revisit the strategies and measures adopted in China to control and eliminate malaria and discuss what can be learnt from its elimination

Malaria is a mosquito-borne infectious disease that significantly threatens global health. Considerable efforts and investments have led to a steady decline in incidence and mortality over recent decades. However, 249 million cases were reported from 85 countries and areas in 2022, resulting in 608 000 deaths.^1^ Notably, approximately 95% of these cases and deaths occurred in the African region. China has had a heavy disease burden of malaria for more than 3000 years, evidenced by the Chinese character for malaria—疟 or nüè—discovered on oracle bone and bronze inscriptions from between 1562 and 1066 BC.^2^ Chinese medicine has historically been used to treat people with malaria. However, in the 1940s, before the foundation of the People’s Republic of China, the burden of malaria was still immense, with an estimated 30 million annual cases, more than 90% of the population at risk, and a fatality rate of approximately 1%.^3^

Amid the grave challenges posed by malaria, China adopted stratified strategies and took various measures to contain the disease. On 14 March 1952 the central government initiated a national health campaign, which called for the public to take action and “eliminate the four pests”—mosquitoes, flies, rats, and sparrows—with mosquito control being one of the core goals. The efforts against mosquitoes’ primary habitats and reproductive cycles greatly benefited public hygiene, promoted health knowledge, and played an important role in controlling infectious diseases, including malaria. Meanwhile, a series of malaria related laws and regulations were issued to guide malaria control practices. Following several decades of concerted efforts nationwide, the World Health Organization certified China as malaria-free on 30 June 2021,^4^ a remarkable achievement for China and a major milestone of global malaria eradication efforts.

As a mosquito-borne disease, transmission of malaria involves multiple factors, including vectors (*Anopheles* mosquitoes), pathogens (*Plasmodium*), hosts (human and animal hosts), and environmental conditions (for example, temperature, precipitation), which complicates its control. Considerable efforts have been made to target *Plasmodium*, such as rapid diagnosis and treatment (for example, artemisinin)^5^ and mass drug administration. However, vector control plays the most important role in blocking malaria transmission, as *Plasmodium* relies on mosquitoes to complete its life cycle and mosquito bites to infect humans. Effective malaria control is heavily dependent on our ability to control mosquito populations.^6^

Although a vast literature is dedicated to elimination of malaria,^7^ the significance of vector control is scarcely revisited in China’s malaria elimination. Therefore, this article, part of a BMJ collection on malaria control in China, revisits China’s seven decade endeavour to control vectors for malaria elimination nationwide. Other articles in this collection explore interventions in specific provinces with distinct conditions over different periods. These include the border province of Yunnan (diverse ecological features, multiple vector species, and underdeveloped economics) since 1980 and the tropical province of Hainan (warm, humid climate year round, with a high volume of tourists and migrant workers) from 1959 to 2011. These national and distinct regional experiences offer valuable insights for countries facing similar challenges. The collection also delves into responses to resurgence of malaria after local elimination and discusses innovative methods for controlling mosquito larvae, aiming to provide a comprehensive view of China’s vector control journey for malaria elimination.

## Evolving malaria vector control strategies in China

**Fig 1:**
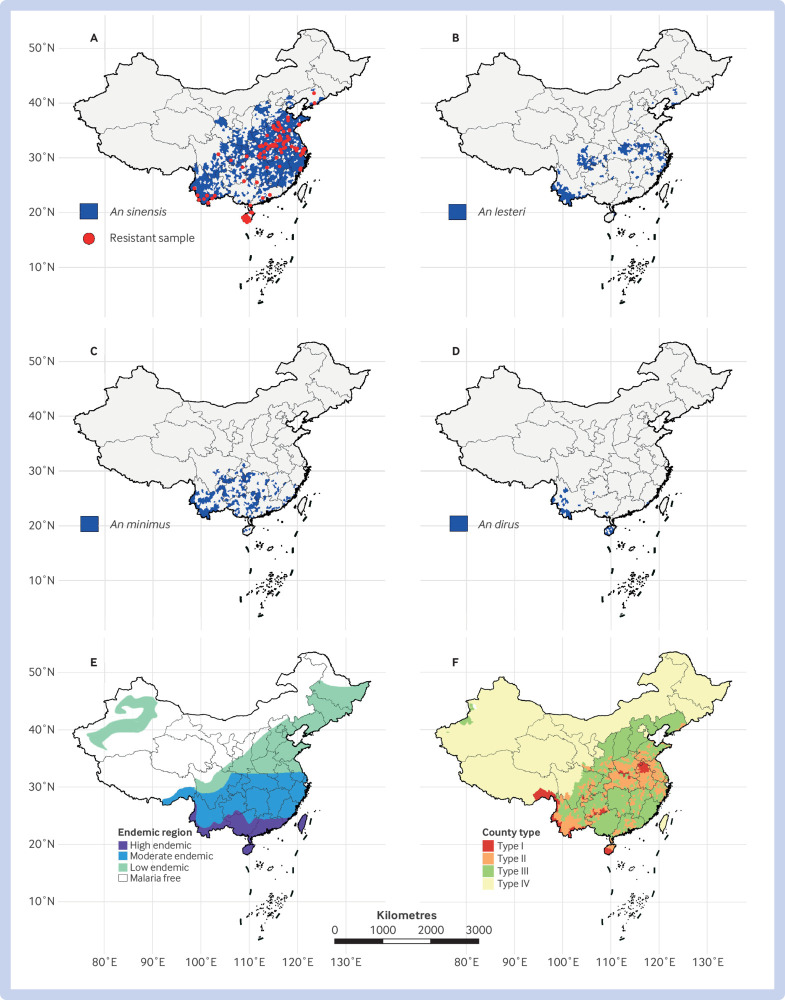
Geographic distribution of four major malaria *Anopheles* in mainland China between 2000 and 2016: *An sinensis* (A), *An lesteri* (B), *An minimus* (C), and *An dirus* (D).^8^ Red dots in map A indicate that insecticide resistance has been reported in *An sinensis* population (data from https://apps.who.int/malaria/maps/threats/). Stratified malaria endemic regions in 1979 (E) and 2010 (F). County type I=annual incidence >10/100 000, with indigenous cases reported in three consecutive years; type II= indigenous cases reported in past three years but annual incidence <10/100 000 in at least one year; type III=no indigenous cases in past three years; type IV=non-malaria endemic regions

The primary malaria vectors in China are *Anopheles* mosquitoes: *An sinensis*, *An lesteri*, *An minimus*, and *An dirus* (fig 1, panels A-D).^9^ Among these, *An sinensis* is the most widespread and dominant species.^10^ *An lesteri*, also known as *An anthropophagus*, acts as the main vector for *Plasmodium falciparum* malaria and has a much higher vector competence than *An sinensis*,^10^ although its geographical range has shrunk in recent years; it is now mainly found in southwest and northeast China (for example, Liaoning Province).^11^ *An minimus* thrives south of the 25°N parallel and serves as a primary malaria vector in southern China, whereas *An dirus* can be found in Hainan and Yunnan provinces.

Vector control plays a crucial role in the path towards elimination of malaria, and China has taken various measures to control mosquitoes.^9^ Initial efforts were directed towards the elimination of mosquitoes. In 1953 dichlorodiphenyltrichloroethane (DDT) treated bed nets were introduced for *Anopheles* control in Yunnan Province, marking one of the earliest interventions. In 1976 China conducted its first research on pyrethroid insecticide treated bed nets,^9^ which proved highly effective in preventing mosquito bites.^12^ From 2011 to 2019 more than 2.2 million insecticide treated bed nets/long lasting insecticidal nets were distributed nationwide. Additionally, indoor residual spraying has been widely implemented since 1980 to control malaria vectors in *P falciparum* endemic areas, protecting more than 5.7 million people between 2011 and 2019.^11^ To supplement these efforts, agricultural innovations, such as rice-fish co-culture, have also contributed to effective mosquito larval control.

Vector control strategies for malaria in China have evolved over time. In 1978 China introduced integrated mosquito management, which combined environmental, chemical, biological, and physical control methods to target harmful mosquito species. Integrated mosquito management aimed to reduce vector mosquito density below a risk threshold, to eliminate vectors in certain conditions and some areas, and ultimately to wipe out the mosquitoes and diseases carried by them. The implementation of integrated mosquito management from 1986 to 1988 in Shandong and Guangdong provinces resulted in a reduction in mosquito density of more than 90%.^13^ Subsequently, integrated vector management was proposed in 1980, following similar principles but encompassing a broader range of vector control techniques.

However, with evolving environmental and social factors in the 1990s and 2000s (for example, climate change, globalisation, urbanisation, changes in land use patterns, and insecticide resistance), malaria remained rampant and resurged in some areas. Amid these challenges, sustainable vector management was introduced in 2004, emphasising the importance of timely vector surveillance to conduct practical risk assessments and formulate control plans for vectors and related diseases on the basis of comprehensive health, economic, and ecological benefits. Sustainable vector management also underscored the benefits of public participation and multi-departmental collaboration to maintain vector populations at a level posing minimal threat of harm. Under the guidance of sustainable vector management, vector surveillance was strengthened and the “epidemic focus response” strategy was practised in a timely and accurate manner, to contain malaria transmission by also targeting mosquito vectors around identified malaria hotspots.

## Measures to control malaria in different phases

**Fig 2:**
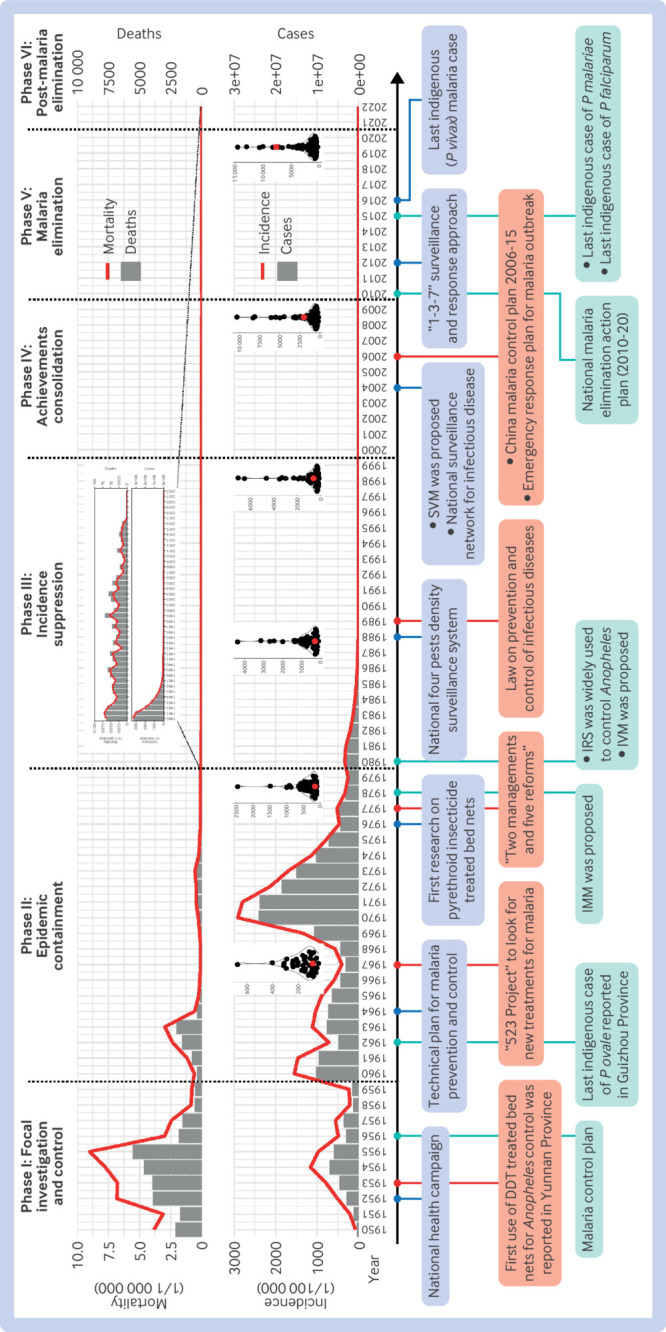
Mortality rate and incidence of malaria in China since 1950, and corresponding measures taken to eliminate malaria. Mini dot plots represent average gross domestic product per capita (US$) of 54 African countries (black dots) and China (red dot) every 10 years from 1960 to 2019 (data from World Bank: https://data.worldbank.org/indicator/NY.GDP.PCAP.CD). DDT=dichlorodiphenyltrichloroethane; IMM=integrated mosquito management; IRS=indoor residual spraying; IVM=integrated vector management; SVM=sustainable vector management

Since 1949 China has used different strategies and measures to control malaria and its vectors on the basis of epidemic characteristics and socioeconomic development. These practices, including the matter of laws and regulations as well as the stratified malaria control strategy, evolved over six distinct phases, each tackling unique challenges in the fight against malaria (fig 2; box 1).

Box 1 **Summary of temporal changes in malaria prevalence in China**

Malaria was prevalent in China in the 1950s, with a significant mortality rate. In 1950 alone, 2123 malaria related deaths were reported, leading to a mortality rate of 3.85/1 000 000. Various measures were taken in this phase, including a national health campaign to eliminate mosquitoes. By 1959, the incidence had dropped to 234/100 000 and the mortality rate had declined to 0.81/1 000 000.
The incidence of malaria fluctuated significantly during 1960-79, reaching a record high in 1970, resulting from a combination of food shortages, increased population mobility, and unstable political conditions. By 1979, the incidence had dropped to 255/100 000—a 91% decrease compared with that in 1970. Malaria intensity in China continued to decline significantly in the 1980s and 1990s. By 1999, malaria incidence had declined to 2.4/100 000.
However, the Huai River Basin in central China experienced an epidemic of *Plasmodium vivax* malaria from 2001 to 2007, leading to a substantial increase in the prevalence of malaria. After strengthening of control measures, malaria incidence dropped back to 1.1/100 000 by 2009. Since the last indigenous malaria case reported in April 2016 in Yunnan Province, China has been malaria-free. However, with thousands of imported cases each year, the challenge remains.

In phase I (1949-59), the initial malaria control strategy focused on “focal investigation and control” to reduce the incidence and mortality, as well as to curb local outbreaks. To facilitate vector control, the country was stratified into four zones on the basis of *Anopheles* species: zone 1 covered the tropical and subtropical areas (south of 25°N), where *An minimus* served as the main malaria vector in mountainous areas and *An sinensis* in plain areas; zone 2 covered the areas between 25°N and 33°N, where the main vectors were *An lesteri* and *An sinensis*; zone 3 was north of 33°N, where *An sinensis* acted as a dominant vector; zone 4 consisted of malaria-free areas (for example, cold high altitude areas, deserts, and plateaus).^10^ ^14^ ^15^

Malaria was particularly prevalent in phase II (1960-79). In response, four zones were determined according to malaria incidence, from high endemic to malaria-free regions (fig 1, panel E).^14^ Control strategies were also locally tailored according to the vector species. In northern China, where *An sinensis* was the main vector, a comprehensive control strategy focused on eliminating infection sources, supplemented by mosquito control. In southern China, where *An minimus* acted as the primary vector, the strategy equally focused on eliminating infection sources and mosquito control.^16^ In areas with *An dirus* (for example, Hainan Province), environmental transformation projects were conducted to remove mosquito breeding sites.^15^

As malaria was progressively reduced in phase III (1980-99), the stratified zones were updated on the basis of the changing reports of malaria incidence. Areas with an annual incidence rate above 1% or areas with endemic *P falciparum* malaria were prioritised and control measures were strengthened in these areas. In areas where the incidence rate dropped below 0.05%, the focus shifted to malaria surveillance.

During phase IV (2000-09), in response to the resurgence of malaria and challenges such as insecticide resistance, sustainable vector management was proposed in 2004 to guide vector control. For example, in Yongcheng of Henan Province, where an outbreak occurred between 2006 and 2009, *Bacillus sphaericus* was used on *An sinensis* breeding sites to prevent the spread of malaria.^17^ In Yunnan Province, housing structures and materials were found to be associated with malaria prevalence. Replacing traditional housing materials (for example, mud and brick, grass-mud mixture, and mud-wood structures) with modern alternatives (for example, concrete and bricks) could significantly reduce mosquito exposure.^18^

In 2010, following the National Malaria Elimination Action Plan (2010-20), the malaria control strategy focused on both individual malaria cases and epidemic spots, aiming to eliminate malaria nationwide in China. Correspondingly, counties were stratified into four types on the basis of malaria incidence (fig 1, panel F),^15^ with resources allocated accordingly. Most importantly, the “1-3-7” strategy (case reporting within one day, investigation within three days, and response within seven days) was proposed to enhance case based malaria surveillance and response.^4^ ^19^ Meanwhile, a nationwide surveillance network was also established, consisting of well distributed sentinel sites and a laboratory network extending to the county level for diagnosis of malaria since 2010.^20^

Since achieving malaria-free certification in 2021, China has shifted its focus to managing imported cases. Rapid interventions are carried out around the epidemic spot to avoid secondary transmission. For instance, indoor residual spraying is conducted in all houses within a 500 m radius of a confirmed case, and neighbouring residents, particularly those with a fever, have rapid diagnostic tests. Routine pathogen surveillance of mosquito vectors in the field also serves as a crucial proactive measure in preventing the resurgence of malaria in this post-elimination phase.

## Key lessons from China’s malaria elimination journey

After 70 years of concerted efforts, China finally eliminated a disease that had been prevalent for thousands of years. However, progress towards elimination of malaria was arduous and full of setbacks. During the early stages, mosquito control relied mainly on insecticides, but their frequent application led to fatigue among residents, diminishing public cooperation. The ensuing insecticide resistance and environmental pollution further hindered control of mosquitoes. In addition, famines across China from 1959 to 1961 devastated China’s population health and economy, resulting in underinvestment in control of mosquitoes and malaria. Meanwhile, the increased population mobility and the dissolution of some disease prevention facilities exacerbated the malaria situation, leading to a large resurgence of malaria in the 1960s. From the late 1960s to the mid-1970s, political turmoil paralysed the national health system, combined with a large influx of susceptible urban populations to malaria endemic rural regions, triggering two major malaria epidemics in central China. In addition, large dry lands were converted into rice paddy fields to boost rice production, which inadvertently created more larva breeding sites and complicated malaria control efforts.

In the 2000s the prolonged period of low malaria prevalence led to complacency and neglect in malaria control. Funding was significantly reduced, skilled personnel were lost, disease prevention facilities were closed, and public awareness of malaria prevention diminished. In some areas, more than 90% of malaria cases went unreported. This neglect led to the re-establishment of malaria in central China. For example, Yongcheng reported its first malaria outbreak in 2003 after 11 years of being malaria-free, accumulating more than 6500 cases from 2006 to 2010. These setbacks underscore critical lessons in the importance of sustained vigilance and investment in fighting against malaria.

Despite these challenges, China ultimately brought malaria under control and achieved elimination. Some previous studies have discussed different aspects of China’s malaria elimination.^7^ ^10^ ^16^ ^21^ The factors contributing to the successful elimination are multifaceted, including strong political commitment and leadership,^22^ ^23^ public involvement (for example, national health campaign), sufficient financial support (for example, funding from the Global Fund to Fight AIDS, Tuberculosis and Malaria),^20^ strengthened surveillance, rapid diagnosis and case management (for example, the “1-3-7” strategy), and timely assessment and supervision. However, vector control is an indispensable component of this success.^6^ Owing to a lack of sufficient insecticides, vector control in the early stage primarily relied on environmental management. When a large volume of insecticide was obtainable, vector control shifted to massive application of insecticide, which inevitably led to severe insecticide resistance and environmental pollution. To cope with these problems, integrated mosquito management and sustainable vector management were proposed and practised in China’s vector control, underscoring the importance of vector surveillance to maintain low vector populations. Meanwhile, the stratified sustainable vector control also played a key role, which is particularly important for countries with diverse ecological, social, and vector species variations. Control measures were continuously carried out to target vector larvae (for example, the removal of breeding sites, and larviciding) or adults (for example, indoor residual spraying, insecticide treated bed nets/long lasting insecticidal nets). Innovative interventions also contributed, such as rice-fish co-culture to suppress larval breeding and replacing traditional housing materials with modern alternatives to reduce risks of exposure to mosquitoes.

## Perspective

Elimination of malaria does not mark the endpoint for control of this disease, but it marks a new beginning for China. Climate change may drive the expansion of mosquito habitat and vector distribution.^24^ Such an expansion could increase the risk of spreading malaria, particularly in historically non-endemic regions. Moreover, insecticide resistance is an imminent problem and may undermine the effectiveness of mosquito control (fig 1, panel A).^25^ Resistance to antimalarial drugs is another non-negligible challenge in the treatment of malaria.^10^

More importantly, the elimination of malaria in China not only benefits domestic public health but also has a broader significance globally. Considering the evolving economic and social conditions over the past 70 years and the diverse environmental and ecological contexts across China, the strategies and measures adopted in China could provide useful information to other countries and regions still grappling with malaria, particularly for the African region. Although malaria is considered a poverty related disease and associated with inadequate economic development,^26^ this does not necessarily mean that malaria could not be controlled or even eliminated in less developed countries. In China’s case, the gross domestic product per capita was comparable to those of many African countries before the 1990s but malaria was still brought under control (fig 2). Through initiatives such as the Forum on China-Africa Cooperation (FOCAC), China has launched a series of malaria control projects with African countries since 2000.^27^ For instance, launching mass drug administration in Comoros in 2007 led to malaria cases being reduced by 95%.^27^ The China-UK-Tanzania Pilot Project conducted between 2015 and 2018 achieved an 81% reduction in malaria prevalence.^28^ This project helped to develop the “1,7-malaria reactive community-based testing and response” (1,7-mRCTR), a locally tailored approach adapted from China’s “1-3-7” strategy, showing a promising effect on malaria control in Tanzania.^28^ Such collaborations offer a platform to share techniques, health products, and intervention strategies in malaria control.^23^ ^29^

China’s journey underscores that elimination of malaria is achievable with sustained political commitment, public involvement, adequate financial support, and strengthened stratified sustainable vector control strategies and measures. The lessons and experiences over the past 70 years are invaluable in advancing global health efforts towards the ultimate goal of malaria eradication worldwide.

**Key messages**

The ongoing extensive national health campaign launched in China in the 1950s has played a crucial role in China’s successful elimination of malaria
Malaria control in China evolved through six phases: focal investigation and control (1949-59), epidemic containment (1960-79), incidence suppression (1980-99), consolidation of achievements (2000-09), malaria elimination (2010-20), and post-malaria elimination (since 2021)
Stratified malaria control strategies have been adopted in different regions, with tailored phases based on disease incidence, vector species, and socioeconomic development
Vector control strategies have evolved over the past seven decades—from eradication of mosquitoes, through integrated mosquito management, to sustainable vector management
Insecticide treated bed nets, indoor residual spraying, and some agricultural innovations have been shown to be effective in controlling mosquitoes and have contributed to elimination of malaria in China

## Notes

Contributors and sources: QL is a leading expert and scientist for vector surveillance and control and leads national research on human health and climate change in China; he also heads the WHO Collaborating Centre for Vector Surveillance and Management. QL contributed to the conceptualisation of the manuscript and supervised the research activity planning and execution. QL and YW (joint first authors) searched the literature, collected the data, and wrote the manuscript with equal contribution. YW produced the figures. XL, SH, and SL discussed the structure and context of the manuscript and provided important comments on the draft manuscript. All authors read and approved the manuscript. The corresponding author confirms that all authors have seen and approved the final text.

Provenance and peer review: Commissioned; externally peer reviewed.

This article is part of a collection developed in partnership with the Chinese Center for Disease Control and Prevention, Beijing. Open access fees were funded by the Gates Foundation, China. *The BMJ* commissioned, peer reviewed, edited, and made the decision to publish. Rachael Hinton and Jocalyn Clark were the lead editors for *The BMJ*.
